# Choroidal vascularity index as a measure of vascular status of the choroid: Measurements in healthy eyes from a population-based study

**DOI:** 10.1038/srep21090

**Published:** 2016-02-12

**Authors:** Rupesh Agrawal, Preeti Gupta, Kara-Anne Tan, Chui Ming Gemmy Cheung, Tien-Yin Wong, Ching-Yu Cheng

**Affiliations:** 1 Singapore Eye Research Institute and Singapore National Eye Center, Singapore; 2 National Healthcare Group Eye Institute, Tan Tock Seng Hospital, Singapore; 3 Department of Ophthalmology, Yong Loo Lin School of Medicine, National University of Singapore and National University Health System, Singapore; 4 Duke-NUS Graduate Medical School, Singapore

## Abstract

The vascularity of the choroid has been implicated in the pathogenesis of various eye diseases. To date, no established quantifiable parameters to estimate vascular status of the choroid exists. Choroidal vascularity index (CVI) may potentially be used to assess vascular status of the choroid. We aimed to establish normative database for CVI and identify factors associated with CVI in healthy eyes. In this population-based study on 345 healthy eyes, choroidal enhanced depth imaging optical coherence tomography scans were segmented by modified image binarization technique. Total subfoveal choroidal area (TCA) was segmented into luminal (LA) and stromal (SA) area. CVI was calculated as the proportion of LA to TCA. Linear regression was used to identify ocular and systemic factors associated with CVI and subfoveal choroidal thickness (SFCT). Subfoveal CVI ranged from 60.07 to 71.27% with a mean value of 65.61 ± 2.33%. CVI was less variable than SFCT (coefficient of variation for CVI was 3.55 vs 40.30 for SFCT). Higher CVI was associated with thicker SFCT, but not associated with most physiological variables. CVI was elucidated as a significant determinant of SFCT. While SFCT was affected by many factors, CVI remained unaffected suggesting CVI to be a more robust marker of choroidal diseases.

The choroid is the vascular layer of eye, with one of the highest blood flow of any tissue in the body^1^. The choroid is predominantly composed of blood vessels surrounded by stromal tissue comprising of connective tissue, melanocytes, nerves and extracellular fluid^2^. The vascular layer of the choroid may be differentiated into 3 layers from internal to external, with increasing luminal diameter. The innermost layer is the choriocapillaris, the middle is Sattler’s layer with medium vessels, and the outer is Haller’s layer, with large vessels^1^.

Being a major vascular layer of the eye the choroid plays an important role in ocular health, and is involved in the pathogenesis of many intraocular diseases such as age-related macular degeneration, polypoidal choroidal vasculopathy, central serous chorioretinopathy and myopic macular degeneration^3^,^4^,^5^,^6^,^7^,^8^,^9^. There is evidence from histological studies that the disease processes affect the stroma and vasculature of the choroid^10^,^11^,^12^. However, shrinkage occurs during the fixation of the tissues during the histological process, making it difficult to study the changes in vascular tone of the choroid^13^.

Choroid thickness (CT) has been reported as an indicator of ocular^14^,^15^,^16^,^17^ and systemic health^18^,^19^,^20^,^21^,^22^. Although, many studies have now reported changes in the thickness of the choroid in various ocular and systemic diseases^8^,^9^,^10^,^11^,^12^ and proposed CT as a marker to assess these disease conditions, there exists a notable disparity in CT in various clinical studies. One such example is diabetes mellitus^23^,^24^,^25^,^26^,^27^, where there has been no consensus as to whether it causes an increase or decrease in CT. This raises the question of what structures in the choroid changes with increasing or decreasing CT and if there is a more robust marker to assess choroid health.

To answer this question, morphological and vascular analyses of the choroid may provide some clues and lead to the development of a more stable marker. The advent of enhanced depth imaging (EDI) optical coherence tomography (OCT) has allowed more precise non-invasive quantitative assessment of the choroid^28^. Using EDI OCT, there have been attempts to assess the choroidal stromal and vascular structures^13^,^29^,^30^,^31^,^32^. Recently Sonoda *et al.* described a method for computing luminal and interstitial areas in the choroid as a means to quantify vascular status of the choroid^30^,^31^.

Adapting the image segmentation technique proposed by Sonoda *et al.*^30^,^31^, we further propose a new quantitative parameter called choroidal vascularity index (CVI) to assess vascular status of the choroid through image binarization of EDI SD-OCT images in healthy eyes. Furthermore, we aimed to determine the ocular and systemic factors affecting the CVI as well as CT in subjects enrolled from a population-based study in Singapore. This index may provide additional information on the morphology and physiology of the choroid and may be a more robust marker compared to CT.

## Methods

### Study population

The data for this study was derived from the Singapore Malay Eye Study-2 (SiMES-2), a population based cohort study of 45–85 years old Malay adults living in Singapore. This study was conducted as per the tenets set forth in the Declaration of Helsinki, and ethics committee approval was obtained from the Institutional Review Board of Singapore Eye Research Institute. Written informed consent was obtained from the subjects after explanation about the details of the study and any potential risks involved with the study and consequences of the study.

### Study subjects

Details of the study design, and methodology have been reported elsewhere^33^. In this study, we enrolled 400 consecutive participants from February 2012 to April 2013. Exclusion criteria included: logarithmic minimum angle of resolution (logMAR) visual acuity >0.30, spherical equivalent (SE) <−6 diopter, evidence of vitreo-retinal diseases such as age related macular degeneration and diabetic retinopathy, previous ocular surgery or clinical features compatible with a diagnosis of glaucoma and Spectralis OCT imaging with a quality index <18 decibels. Glaucoma was defined using the International Society of Geographic and Epidemiological Ophthalmology scheme^34^, based on findings from gonioscopy, optic disc characteristics, and visual fields results.

### Choroidal thickness assessment

The choroid was imaged using the EDI mode of SD-OCT (Spectralis, Heidelberg Engineering, Heidelberg, Germany). The macular region was scanned using a 7 horizontal line scan (30° × 5°) centred on the fovea, with 100 frames averaged in each B-scan. Each scan was 8.9 mm in length and spaced 240 μm apart from each other. In our study, Bruch’s membrane and the choroid-scleral interface were delineated with the automatic segmentation algorithm developed by Tian *et al.*^35^ which demonstrated excellent repeatability in our previously reported population-based study^36^. The choroidal thickness was automatically measured as the distance between the Bruch’s membrane (lower boundary of retinal pigmented epithelium [RPE]) and the choroid-scleral interface. Although measurements of both eyes of each study participant were obtained, due to inter eye correlation only the right eye was used for further analysis.

### Image binarization details

**Figure 1:**
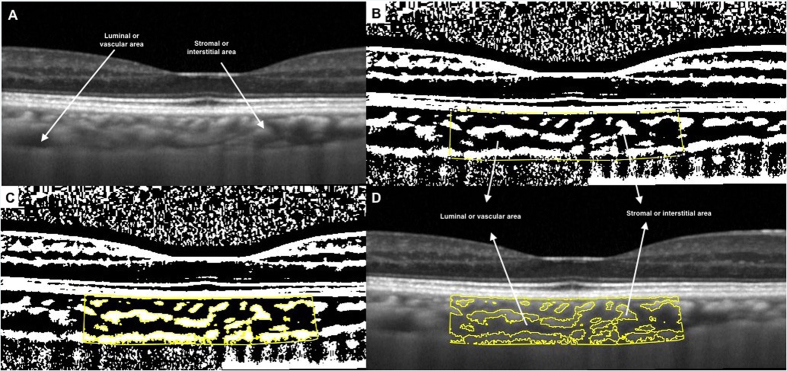
Image binarization for choroid with normal choroidal thickness. (**A**) Original SD OCT image. (**B**) 1.5 mm segmentation block of the subfoveal choroidal area. (**C**) Segmented OCT image using modified image binarization approach. (**D**) Overlay of region of interest created after image binarization was performed on the SD OCT image.

The same raster scan passing through the fovea was selected for binarization. It was segmented using the protocol described by Sonoda *et al.*^30^,^31^ with minor modifications. The image binarization was done using public domain software, Image J (version 1.47; http://imagej.nih.gov/ij/). The subfoveal choroidal area with a width of 1.5 mm, centred at the fovea, was selected (Fig. 1A) and this constituted the region of interest. Only 1.5 mm of the macular area on the single line scan was selected as a representative segment of the macular region due to segmental nature of the choroidal blood supply as described by Hayreh^37^. The posterior ciliary arteries and their branches along with terminal choroidal arterioles, the choriocapillaris, and the vortex veins have a segmental or lobular distribution in the choroid.

Image binarization techniques can be used to convert grey scale images into binarized images. This facilitates tasks such as image layout analysis and image skew estimation. An appropriate image binarization technique, taking into account the uneven illumination, image contrast variation and poor image resolution, is essential to accurately apply a threshold to an image. Different image binarization or thresholding techniques like Otsu’s, Bernsen’s and Niblack’s autolocal thresholding techniques were hence attempted^38^,^39^. Otsu’s is a global thresholding technique while Bernsen’s is local thresholding technique. After comparing the different image segmentation techniques, we adopted Niblack’s autolocal threshold technique in our current study. This is because it takes into consideration the mean and standard deviation of all the pixels in the region of interest. In addition, given that binarization could be influenced by the variation in the amount of melanin in RPE in different eyes, and also affected by the direction of light and focussing issues, these were taken into account by using a distinct binarization threshold for individual subject.

Using Niblack’s autolocal threshold tool, the image was first binarized to get a clear view of the choroid-scleral interface (Fig. 1B). This was to allow more precise selection of the subfoveal choroid area. This is in contrast to Sonoda’s *et al.*^30^,^31^ protocol in which the polygonal area was selected prior to image binarization. In addition, we did not preselect vessels of size more than 100 um.

With the upper border marked at the RPE and the lower border the line of light pixels at the choroid scleral junction, the choroidal area was selected using polygon tool and added to the region of interest manager (Fig. 1B). The image was then converted to RGB (red, green, blue) colour to allow the colour threshold tool to select the dark pixels (Fig. 1C). The total subfoveal circumscribed choroidal area (TCA) and the area of dark pixels were calculated. The luminal area (LA) was defined as the area of dark pixels. Stromal area (SA) was further calculated by subtracting LA from TCA. To determine the vascularity status of the choroid, CVI was computed by dividing LA by TCA. In addition, the proportion of dark (LA) to light areas (SA) was also computed. Fig. 1D represents the overlay image of the region on interest on the original EDI OCT scan.

### Inter-rater and Intra-rater agreement

10% of the total images (35 images), were initially segmented by two graders (KAT and RA) to determine inter-rater agreement. The same set of images was segmented by one grader (KAT) after an interval of one week to compute intra-rater reliability. The intra- and inter-rater reliability for the image binarization was measured by the absolute agreement model of the intra-class correlation coefficient (ICC)^40^. ICC value of 0.81–1.00 indicates good agreement. Values of less than 0.40 indicate poor to fair agreement. We also performed Bland-Altman plot analyses^41^,^42^ to determine the mean difference between the measurements. The Bland-Altman plots were constructed using MedCalc version 12.3 (Medcalc Software, Ostend, Belgium) software. Moreover, random scans, including those with thick and thin choroid, were further reviewed by both graders to ensure good inter-rater agreement. After obtaining good inter-rater and intra-rater agreement, all the scans were binarized by single author (KAT).

### Measurement of ocular factors

Each participant underwent a standardized examination. Refraction and corneal curvature were measured using an auto-keratorefractor (Canon RK 5 Auto Ref-Keratometer, Canon Inc. Ltd., Tochigiken, Japan). SE was calculated as the sum of the spherical power and half of the cylinder power. Best-corrected visual acuity was measured monocularly using a LogMAR chart (Lighthouse International, New York, USA) at a distance of 4 meters. Ocular biometry, including axial length (AL), was measured using non-contact partial coherence interferometry (IOL Master V3.01, Carl Zeiss Meditec AG, Jena, Germany). Intraocular pressure (IOP) was measured using Goldmann applanation tonometry (Haag-Streit, Bern, Switzerland) before pupil dilation. Standardized visual field testing was performed with static automated white-on-white threshold perimetry (SITA Fast 24-2, Humphrey Field Analyzer II; Carl Zeiss Meditec, Inc., Oberkochen, Germany). Slit-lamp biomicroscopy (Haag-Streit model BQ-900; Haag-Streit, Switzerland) was performed by the study ophthalmologists to examine the anterior chamber and lens after pupil dilation with tropicamide 1% and phenylephrine hydrochloride 2.5%.

### Measurement of systemic factors

A detailed interviewer-administered questionnaire was used to collect demographic data, lifestyle risk factors (e.g. smoking, alcohol consumption), medical history (e.g. hypertension, diabetes), ocular history (e.g. glaucoma), and medication use from all participants. Systolic and diastolic blood pressures (BP) were measured using a digital automatic blood pressure monitor (Dinamap model Pro Series DP110X-RW, 100V2; GE Medical Systems Information Technologies, Inc., Milwaukee, WI), after subjects were seated for at least five minutes. BP was measured twice, with measurements 5 minutes apart. A third measurement was taken if the previous 2 systolic BP readings differed by more than 10 mmHg or the diastolic BP differed by more than 5 mmHg. The mean of the two closest BP readings was taken as each participant’s BP.

Mean ocular perfusion pressure (OPP) was calculated using the following equation: mean OPP = (2/3 × mean arterial pressure [MAP] – IOP), where MAP = diastolic BP + (1/3 × [systolic BP – diastolic BP]). Body mass index (BMI) was calculated as body weight (in kilograms) divided by body height (in meters) squared. Smoking status was defined as those currently smoking, ex-smokers and non-smokers. Nonfasting venous blood samples were analysed at the National University Hospital Reference Laboratory for biochemical testing of serum total cholesterol, triglycerides, glycosylated haemoglobin (HbA1c), serum glucose level and creatinine.

### Statistical methods

Statistical analysis was performed using SPSS version 20.0 (SPSS, Inc., Chicago, IL, USA). Since CVI and subfoveal choroidal thickness (SFCT) have different measurement units, we used the coefficient of variation (COV) to compare the variability between CVI and SFCT. Univariate and multiple linear regression analyses were performed to determine the associations of SFCT and CVI (dependent variables) with ocular and systemic factors (independent variables). For multiple linear regression, factors showing significant association in univariate analysis (p < 0.10) were included. All p values were 2-sided and considered statistically significant when the values were less than 0.05.

## Results

A total of 400 subjects were recruited for this study. We excluded 55 subjects for the following reasons: visual acuity worse than 0.30 (n = 9), SE < −6 diopter (n = 7), glaucoma (n = 6), presence of macular or vitreo-retinal diseases (n = 18) and poor OCT image quality (n = 15). A total of 345 eyes from 345 subjects were included in the final analysis; 190 (55%) subjects were female. The demographics, ocular, systemic and choroidal characteristics of the study subjects are shown in Table 1. In terms of choroidal characteristics, mean TCA was 0.74 ± 0.21 mm^2^ and mean LA was 0.49 ± 0.15 mm^2^. Mean SFCT was 241.34 ± 97.11 μm (range, 40.24–519.48 μm) and mean CVI was 65.61 ± 2.33% (range, 60.07–71.27%).

**Figure 2:**
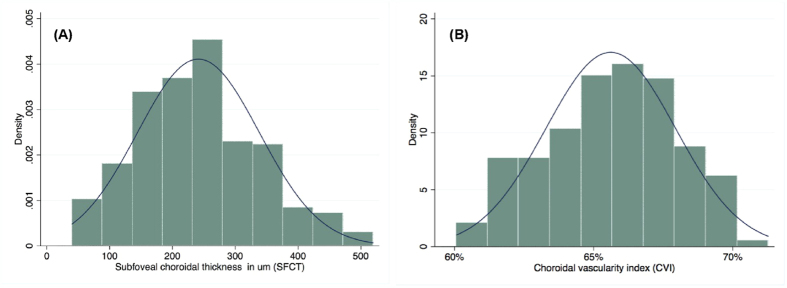
Distribution of subfoveal choroidal thickness (**A**) and choroidal vascularity index (**B**) across the population.

CVI was found to have lower COV (3.55) than SFCT (COV = 40.30), indicating CVI to be less variable than SFCT. The histogram plots (Fig. 2A,B) represent the distribution of SFCT and CVI in relation to normal density plot.

**Figure 3:**
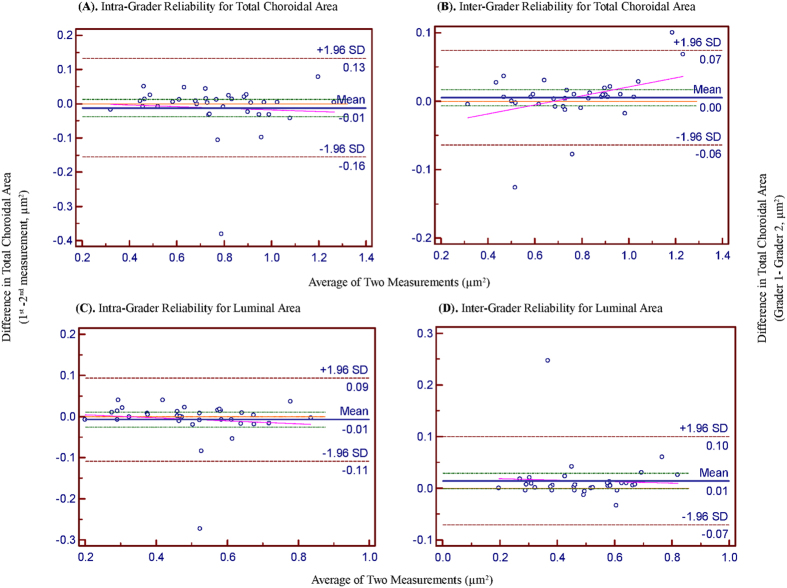
Bland Altman plots of total choroidal area and luminal area. (**A**) and (**B**) shows intra- and inter- rater reliability for total choroidal area respectively. (**C**) and (**D**) shows intra- and inter- rater reliability for luminal area respectively. For intra-rater reliability, the difference was calculated by the 1st measurement minus the 2nd measurement. Pink dashed line represents regression line of difference between 1st and 2nd measurements. For inter-rater reliability, the difference was calculated by the grader 1 measurement minus the grader 2 measurement. Pink dashed line represents regression line of difference between the two graders measurements.

Using image binarization, the intra- (ICC: 0.97 to 0.99 for TCA and ICC: 0.91 to 0.98 for LA) and inter-grader reliability (ICC: 0.90 to 0.97 for TCA and ICC: 0.89 to 0.97 for LA) were excellent for both TCA and LA (Table 2). Bland Altman plot analysis of intra- and inter-rater reliability for TCA (Fig. 3A,B) and LA (Fig. 3C,D) at sub-foveal location was excellent.

In Table 3, the multiple regression model shows younger age, shorter AL, higher IOP, higher LA and lower systolic blood pressure to be significantly (p < 0.05) associated with thicker sub-foveal choroid. However, among factors associated with CVI (Table 4), in the multiple regression model, SFCT was the only factor associated with CVI. A thicker sub-foveal choroid was significantly (p < 0.001) associated with higher CVI. There were no other statistically significant association between CVI and any other factors (Table 4).

## Discussion

In this population-based study, using the modified Sonoda’s image binarization technique for EDI SD-OCT scans, we propose an OCT based metric termed “CVI” to assess vascularity of the choroid. Our results validated the findings obtained by Sonoda *et al.* and found that on a single cross sectional scan, nearly two third (~66%) of the subfoveal choroid is vascular in healthy eyes. Importantly, CVI showed lesser variability and was influenced by fewer physiologic factors as opposed to CT, indicating CVI to be a relatively stable index for studying the changes in the choroid. As the choroid is primarily a vascular structure, understanding of this new vascular index may help to further elucidate the role of vascular processes within the choroid in disease development and progression. We hence propose CVI as an independent surrogate marker to assess choroidal health in future studies.

Several studies have assessed the vascular structures of the choroid by OCT^13^,^29^, but they required customized software that limited their widespread use. There are reports^30^,^31^ on the differentiation and quantification of the structural components of the choroid (luminal and interstitial areas), using freely and easily accessible software, *Image J,* these studies were performed in clinic-based settings with a potential selection and sampling biases. We have highlighted the significant differences in our protocol with that proposed by Sonoda *et al.*^30^,^31^ in Table 5. Our modification of applying auto local threshold prior to image binarization enabled us to accurately localise choroid scleral interface giving more precise selection of the choroid. In addition, the simple binarization technique without pre-selection of larger choroidal vessels allowed nearly accurate estimation of vascularity of the choroid even with a very simple algorithm, which can be reproduced by the large research community.

Although there is no concrete evidence that the dark areas represented the vascular areas and the light areas the stromal areas, the findings of earlier studies and that of numerous empirical observations suggest that the dark areas were the vascular components in the binarized images^13^,^29^. In addition, a comparison of the original EDI-OCT images to the binary images (Fig. 1) revealed that the dark areas corresponded with vascular components of the choroid, including both the larger and smaller choroidal vessels. Therefore, the binarization technique developed by Sonoda *et al.*^30^,^31^, which was further simplified by us, is valid and offers precise segmentation of choroidal vasculature from stroma.

Interestingly, when comparing the factors affecting SFCT to those that affect CVI, we found SFCT to be associated with many physiological factors such as age, AL, IOP and, most significantly, the vascular area in the choroid (LA), whereas stromal area did not have a significant association with SFCT. On the other hand, CVI was only affected by SFCT, but was not affected by most of the physiological variables. Moreover, SFCT demonstrated relatively greater variability (mean SFCT was 241.34 ± 97.11 μm, COV = 40.30) compared to CVI (mean CVI was 65.61 ± 2.33%, COV = 3.55). Thus our results suggest CVI to be a better and relatively more stable marker to monitor choroid compared to CT, which is affected by more variables and demonstrated greater variability. Clinically, measuring the proportion of vascularity of the eye would provide us with a deeper understanding of how disease processes affect different structures in the eye, and therefore may be more informative compared to CT measurements alone.

We have demonstrated a significant association of SFCT with vascular area of the choroid. This signifies the fact that the vascular area is the predominant segment influencing the CT in normal population. An increase in CVI reflects either an increase in the number of blood vessels or in the diameter of the choroidal blood vessels within a designated area. Hayreh^37^ demonstrated the vulnerability of submacular choroidal supply to generalised chronic ischaemic disorders (age related macular degeneration), due to numerous watershed zones of the short posterior ciliary arteries in the choroid. There can be potential clinical implications of the CVI, which can be explored in further studies. A decrease in CVI on EDI OCT scans at baseline may be an indicator of choroidal ischemia in patients with macular disorders like age related macular degeneration or diabetes. On the other hand, we may use CVI to determine increase in vascularity of the choroid in posterior uveitis or central serous chorioretinopathy. CVI can also be used as a follow up tool for treatment response and resolution of diseases.

The strengths of our study include a large sample size with a single common ethnicity. Hence, our findings were unlikely to be confounded by ethnic heterogeneity. Standardized clinical examination protocols, as well as reliable differentiation and quantifications of choroidal morphometric parameters and OCT parameters were used in our study. Nevertheless, this study has few limitations. First, binarization of choroidal images was performed only in the right eye of each study subject. There may exist inter-eye differences, yet such differences should be small. Second, the CT measurements in our study were not performed at the same time of the day; each participant underwent the OCT examination in a randomized manner with respect to when the readings were obtained. It seems unlikely that circadian changes may have influenced the results of our investigation. Third, although our images were binarized at standard threshold, there is a possibility of over or underestimation of both SA and LA.

In conclusion, in this population-based study, we introduced a novel OCT based marker termed “CVI” to assess vascularity of the subfoveal choroid. Our result showed that on a single cross sectional EDI-OCT image, two-third (~66%) of the subfoveal choroid is vascular in healthy eyes. This index should provide a new means of studying the pathophysiology of human choroid in greater detail. However, larger studies for different disease models are warranted to further validate the application of this index in clinical practice. Whether CVI is a complimentary or substitute tool to CT can only be answered based on the proposed studies in choroidal diseases.

## Additional Information

**How to cite this article**: Agrawal, R. *et al.* Choroidal vascularity index as a measure of vascular status of the choroid: Measurements in healthy eyes from a population-based study. *Sci. Rep.* **6**, 21090; doi: 10.1038/srep21090 (2016).

## Figures and Tables

**Table 1:** Demographics, clinical and choroidal characteristics of study subjects (n = 345).

Characteristics	Mean ± SD	Range
Age, yrs	61.53 ± 8.77	47.19 to 86.72
Gender, male (%)	155 (44.9%)	
Axial length, mm	23.58 ± 0.96	21.60 to 27.89
IOP, mm Hg	14.41 ± 2.84	6 to 20
Ocular perfusion pressure, mmHg	55.68 ± 8.39	37.44 to 96.11
Systolic blood pressure, mmHg	139.21 ± 20.63	95 to 226.50
Diastolic blood pressure mmHg	77.27 ± 10.85	54.50 to 133
Body mass index, kg/m^2^	26.75± 5.13	12.15 to 52.95
Serum glucose, mmol/L	6.98 ± 3.35	2.8 to 22.9
HbA1c, %	6.26 ± 1.27	4.3 to 11.7
Total cholesterol, mmol/L	5.43 ± 1.26	2.49 to 10.40
Triglycerides, mmol/L	1.95 ± 1.38	0.42 to 14.95
Blood creatinine, mmol/L	78.92 ± 33.11	30 to 412
Current smoking, %	68 (19.8%)	
Alcohol consumption, %	5 (1.5%)	
Choroidal Parameters		
TCA, mm^2^	0.74 ± 0.21	0.198 to 1.237
LA, mm^2^	0.49 ± 0.15	0.122 to 0.817
SA, mm^2^	0.25 ± 0.06	0.076 to 0.441
CVI (LA/TCA)	65.61 ± 2.33	60.07 to 71.27
LA/SA	1.92 ± 0.20	1.50, 2.48
SFCT, μm	241.34 ± 97.11	40.24, 519.48

Data presented are means ± standard deviations, except for gender, HbA1c , current smoking and alcohol consumption which are number (%).

TCA, total sub-foveal choroidal area; LA, luminal area; SA, stromal area; CVI, choroidal vascularity index; LA/SA, luminal area/ stromal area; SFCT, sub-foveal choroidal thickness.

**Table 2:** Intra- and inter-grader reliability assessment of choroidal parameters in 35 subjects.

	Intra Rater		Intra Rater	
	ICC (95% CI)	Mean difference (95% LOA)	ICC (95% CI)	Mean difference (95% LOA)
TCA	0.99 (0.97 to 0.99)	−0.01 (−0.16 to 0.13)	0.94 (0.90 to 0.97)	0 (−0.06 to 0.07)
LA	0.96 (0.91 to 0.98)	−0.01 (−0.11 to 0.09)	0.94 (0.89 to 0.97)	0.01 (−0.07 to 0.10)

TCA, total sub-foveal choroidal area; LA, luminal area; ICC, intraclass correlation coefficient; CI, confidence interval; LOA, limits of agreement.

**Table 3:** Linear regression analyses of ocular and systemic factors associated with sub-foveal choroidal thickness.

	Univariate			Multivariate^*^		
	Unstandardized β	Standardized β	P-value	Unstandardized β	Standardized β	P-value
Ocular factors						
Axial length, mm	−15.348	−0.155	0.005	−10.508	−0.105	0.002
IOP, mmHg	3.160	0.092	0.087	3.619	0.105	0.002
OPP, mm Hg	−0.354	-0.031	0.572	–	–	–
LA, mm^2^	523.214	0.814	<0.001	459.729	0.714	<0.001
SA, mm^2^	1132.841	0.787	<0.001	68.271	0.047	0.669
Systemic factors						
Age, yrs	−3.986	−0.360	<0.001	−0.856	−0.077	0.041
Gender, female	−22.274	−0.114	0.034	−13.171	−0.068	0.068
Body mass index, kg/m^2^	−1.173	−0.062	0.252	–	–	–
Systolic blood pressure, mmHg	−0.499	−0106	0.050	−0.328	−0.071	0.043
Diastolic blood pressure mmHg	0.749	0.084	0.122	–	–	–
Serum glucose, mmol/L	0.102	0.004	0.949	−	−	−
HbA1c, %	5.913	0.077	0.157	−	−	−
Total cholesterol, mmol/L	8.092	0.106	0.052	−0.525	−0.007	0.836
Triglycerides, mmol/L	3.456	0.049	0.366	–	–	–
Blood creatinine, mmol/L	-0.050	-0.017	0.754	–	–	–
Current smoking, (yes vs no)	28.392	0.116	0.031	−11.737	−0.049	0.187
Alcohol consumption, (yes vs no)	−19.833	0.024	0.651	–	–	–

^*^Adjusted for variables with a p-value < 0.10 in the univariate analysis. β, regression coefficient.

**Table 4:** Linear regression analyses of ocular and systemic factors associated with choroidal vascularity index.

	Univariate			Multivariate*		
	Unstandardized β	Standardized β	P-value	Unstandardized β	Standardized β	P-value
Ocular factors						
Axial length, mm	−0.274	−0.113	0.039	−0.106	−0.004	0.375
IOP, mmHg	−0.014	−0.018	0.745	–	–	–
OPP, mmHg	0.022	0.080	0.136	–	–	–
SFCT, μm	0.012	0.519	<0.001	0.012	0.484	<0.001
Systemic factors						
Age, yrs	−0.057	−0.215	<0.001	−0.001	−0.003	0.950
Gender, female	−0.706	−0.151	0.005	−0.183	−0.039	0.482
Body mass index, kg/m^2^	−0.026	−0.058	0.283	–	–	–
Systolic blood pressure, mmHg	−0.001	−0.012	0.818	–	–	–
Diastolic blood pressure mmHg	0.031	0.145	0.007	0.019	0.089	0.072
Serum glucose, mmol/L	0.025	0.036	0.511	–	–	–
HbA1c, %	0.034	0.018	0.736	–	–	–
Total cholesterol, mmol/L	0.115	0.062	0.256	–	–	–
Triglycerides, mmol/L	0.130	0.077	0.158	–	–	–
Blood creatinine, mmol/L	0.003	0.035	0.516	–	–	–
Current smoking, (yes vs no)	1.098	0.187	<0.001	0.535	0.092	0.088
Alcohol consumption, (yes vs no)	1.004	0.051	0.341	–	–	–

*Adjusted for variables with a p-value < 0.10 in the univariate analysis.

β, regression coefficient.

**Table 5:** Image binarization protocol in Sonoda *et al.* vs current study.

	Sonoda *et al*. (2014)^30^	Sonoda *et al.* (2015)^31^	Current study
Study sample size	15 eyes of 15 subjects	180 eyes of 180 subjects	345 eyes of 345 subjects
Choroidal area measured	1.5 mm	7.5 mm	1.5 mm
Location of measurement	Centered on fovea, 1500 um	Entire raster scan, 7500 um	Centered on fovea (1500 um) due to the segmental nature of choroidal blood supply as described by Hayreh *et al.*^37^
Pre-selection of vessels	3 choroidal vessels with lumens >100 mm were randomly selected and the average reflectivity of these areas was determined by the software		Used autolocal threshold techniques to allow binarization of smaller choroidal vessels or choriocapillaris.
Brightness adjustment	Average brightness was set as the minimum value		Brightness was not adjusted as it would reduce the contrast between luminal and stromal areas and possibly affect the autolocal threshold.
Order of binarization	Region of interest selected prior to image binarization		To get a clear view of the choroid-scleral interface, image binarization was performed prior to area selection.
Image segmentation time	~5 minutes per image		~1 minute per image
